# Study on the Chemical Composition, Biological Activity, and Mechanism of Intervention in Erectile Dysfunction of Healthy Snow Lotus Wine

**DOI:** 10.1002/fsn3.70279

**Published:** 2025-05-19

**Authors:** Bohan Yang, Siyu Tao, Linyang Wang, Yanna Yao, Xinyu Wang, Shuge Tian

**Affiliations:** ^1^ College of Traditional Chinese Medicine Xinjiang Medical University Urumq Xinjiang China; ^2^ Xinjiang Tianshan Snow Lotus Pharmaceutical co., Ltd. Changji Xinjiang China

**Keywords:** biological activity, chemical composition, erectile dysfunction, Healthy Snow Lotus Wine, UPLC–MS

## Abstract

The objective of this study was to investigate the chemical composition, biological activity, and intervention mechanism of Healthy Snow Lotus Wine in the treatment of erectile dysfunction (ED). A preliminary relative characterization and relative quantification of Healthy Snow Lotus Wine was conducted using ultra performance liquid chromatography‐quadrupole orbitrap mass spectrometry (UPLC‐Q‐Orbitrap‐MS). This analysis led to the screening of chemical components that met the criteria of drug similarity and bioavailability. Network pharmacology methods were utilized to identify the active ingredients and their molecular targets of Healthy Snow Lotus Wine that are relevant to the treatment of ED. Enrichment analysis revealed a significant association between Healthy Snow Lotus Wine and pathways related to chemical stress, particularly those involved in cellular basement membrane and phosphatidylinositol 3‐kinase complex signaling. Molecular docking experiments demonstrated that the key compounds bind well to key ED‐related proteins, including STAT3, EGFR, SRC, AKT1, and BCL2. Antioxidant experiments demonstrated that the wine exhibited high antioxidant capacity, with its total phenolic content (TPC), total flavonoid content (TFC), and total proanthocyanidin content (TAC) being superior to those of other samples. These findings provide substantial evidence to support the therapeutic potential of Healthy Snow Lotus Wine in treating ED through multiple mechanisms, underscoring its notable biological activity.

## Graphical abstract

This study investigates the chemical composition, bioactivity, and mechanisms of Healthy Snow Lotus Wine in treating erectile dysfunction (ED). Using UPLC‐Q‐Orbitrap‐MS and network pharmacology, key metabolites and active components were identified, revealing significant antioxidant capacity, lipase inhibition, and multi‐targeted effects on ED‐related proteins. These findings underscore the therapeutic potential of Healthy Snow Lotus Wine for ED treatment through various bioactive pathways.

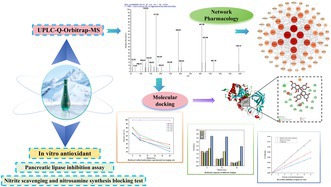

## 1 Introduction

Health Snow Lotus Wine is crafted from high‐quality Xinjiang grapes and infused with traditional Chinese medicinal ingredients, including Tianshan Snow Lotus, wolfberry, longan meat, chrysanthemum, and angelica. Unlike the traditional fermentation method, this innovative product utilizes a modern steeping process to integrate medicinal components into the wine. This approach preserves the wine's original flavor while enhancing its functional properties by dissolving active herbal ingredients into the beverage (Gong et al. 2019). Tianshan snow lotus contains flavonoids, terpenoids, polysaccharides, and other active ingredients (Xie et al. 2017). It has been demonstrated in the scientific literature that Tianshan snow lotus flavonoids and terpenoids possess antibacterial, anti‐inflammatory, analgesic, anti‐tumor, and other biological activities (Báez‐González et al. 2024). Additionally, its polysaccharides possess antioxidant, radioprotective, and photodamage‐mitigating effects. Wolfberry contributes polysaccharides, carotenoids, and flavonoid glycosides, which are known for their immunomodulatory, antifatigue, and ocular neuroprotective activities (Wang et al. 2020). Longan meat, rich in polyphenols and vitamins, supports heart and spleen health while promoting mental calmness and restful sleep (Yue et al. 2022). Chrysanthemum is rich in flavonoids and volatile oils, which can clear away heat and provide detoxification, as well as have antibacterial effects (Liu, Lu, et al. 2024; Liu, Ran, et al. 2024). The ferulic acid and volatile oils in Angelica sinensis are widely used to tonify and invigorate the blood, regulate hormone balance, and fight aging (Wei et al. 2016). The synergistic effect of these herbs further enhances the health and functionality of Healthy Snow Lotus Wine compared to traditional wine, making it an innovative wine product.

Alcohol consumption exhibits a dual impact on erectile dysfunction (ED). Moderate intake may temporarily alleviate ED symptoms through relaxation and vasodilation (Yafi et al. 2016). However, excessive alcohol consumption is a well‐documented risk factor for ED, as it impairs vascular endothelial function, suppresses testosterone production, and exerts toxic effects on the nervous system (S. Li et al. 2021). In contrast, Healthy Snow Lotus Wine, a functional beverage infused with traditional Chinese medicinal ingredients, such as Tianshan Snow Lotus, wolfberry, longan meat, chrysanthemum, and angelica, demonstrates potential benefits for ED management. These ingredients are renowned in traditional medicine for their ability to nourish the liver and kidneys, replenish qi and blood, calm the mind, and alleviate anxiety (Pan et al. 2023). In the meantime, more and more consumers are inclined to treat chronic diseases through the use of medicinal food and food ingredients as a means of health awareness (Arenas‐Jal et al. 2020). As a functional beverage with both medicinal and food properties, Healthy Snow Lotus Wine is gradually gaining popularity among consumers due to its safety and heart acceptance as well as its convenience (Law and Au 2025).

Studies have demonstrated a close association between ED and oxidative stress coupled with metabolic dysregulation. Reactive oxygen species (ROS) impair nitric oxide synthase (eNOS) activity, reducing nitric oxide (NO) bioavailability, and consequently compromising penile vasodilation (Corona et al. 2023; Kaltsas et al. 2024). Additionally, obesity‐induced lipid accumulation exacerbates ED through chronic inflammatory pathways (Subramaniyan and Hanim 2025), and the terpenoids in Healthy Snow Lotus Wine reduce lipid absorption by inhibiting pancreatic lipase activity, thereby alleviating adipose tissue‐mediated inflammatory responses (Rajan et al. 2020). This anti‐inflammatory effect is characterized by decreased levels of pro‐inflammatory cytokines, such as interleukin‐8 (IL‐8) and C‐reactive protein (CRP), ultimately ameliorating the severity of ED (Moon et al. 2019).

In this study, we used UPLC‐Q‐Orbitrap‐MS to characterize the chemical constituents of Healthy Snow Lotus Wine (Kim et al. 2024), combining qualitative and relative quantitative analyses with functional studies to unravel its potential mechanisms for addressing ED. High‐resolution mass spectrometry data were utilized to identify bioactive components and predict their interactions with ED‐related protein targets through molecular docking and network pharmacology. Furthermore, the antioxidant capacity and pancreatic lipase inhibitory activity of the wines were systematically evaluated by standardized in vitro assays, directly targeting oxidative stress and metabolic dysregulation—two key pathways implicated in the pathogenesis of ED. Through phytochemical analysis and bioactivity validation, this study elucidated the relationship between its chemical composition and bioactivity, thus laying a scientific foundation for its application in ED management and functional food development.

## 2 Materials and Methods

### 2.1 Wine Samples

Healthy Snow Lotus Wine produced by Xinjiang Tianshan Snow Lotus Pharmaceutical Co. Ltd. and three other Cabernet Sauvignon wine samples were selected for the determination of total composition and antioxidant testing. W1, W2, and W3 were Cabernet Sauvignon wines, and W4 was the Healthy Snow Lotus Wine. The wine samples were stored in the laboratory at 4°C in a refrigerator and were tested and analyzed immediately after opening the bottles.

### 2.2 Lipase Inhibition Test

#### 2.2.1 Inhibition of Lipase by Red Wine

According to the method in the literature (Pandey et al. 2020; Sosnowska et al. 2022), p‐NPB was predissolved in acetonitrile, and a solution of 5.6 μmol/mL was prepared and stored at −20°C. The solution was prepared in Tris–HCl buffer (13 mmol/L, pH = 7.5) and stored at 4°C. Tris–HCl buffer (13 mmol/L, pH = 7.5) was prepared and stored at 4°C. An appropriate amount of lipase was dissolved in Tris–HCl buffer, centrifuged at 3600 × g for 20 min at 4°C, and the supernatant was collected and prepared into a 0.25 mg/mL lipase solution, stored at 4°C for spare use. Prepare the wine samples at dilutions of 1, 2, 4, 6, and 8, add 50 μL of wine samples at different dilutions into 96‐well plates, add 130 μL of lipase solution, incubate at 37°C for 10 min, and add 20 μL of 5.60 μmol/mL substrate p‐NPB solution, incubate at 37°C for 60 min, and then read absorbance values at 405 nm. In the blank control group, distilled water was used instead of wine samples, Tris–HCl buffer was used instead of lipase solution, and orlistat (0.5 mg/mL) was used as a positive control, and each group of experiments was done three times in parallel, and the inhibition rate was calculated.

$$\text{Inhibition rate}\;\left(\%\right)=\left(1-\frac{C-D}{A-B}\right)\times 100$$

where A represents the Blank group (no sample, enzyme added), B the Blank control group (no sample, no enzyme added), C the sample group (with sample, with enzyme), and D the Sample control group (with sample, without enzyme).

#### 2.2.2 Type of Lipase Inhibition by Red Wine

Referring to the literature (Cao et al. 2024; N. Li et al. 2022), 50 μL of diluted zerofold, undiluted, and twofold wine samples were taken from a 96‐well plate and added with 130 μL of 0, 2, 3, 4, 5, and 6 mg/mL lipase solution, respectively, then incubated at 37°C for 10 min, and then added with 20 μL of 5.60 μmol/mL of the substrate, p‐NPB, and the absorbance was read at 405 nm after incubation for 60 min at 37°C. The absorbance value was read at 405 nm after incubation at 37°C for 60 min. The wine samples with a dilution of 0 were replaced by distilled water, and the 0 mg/mL lipase solution was replaced by Tris–HCl buffer, and each set of experiments was done three times in parallel (Zhang et al. 2025).

According to the above procedure, the concentration of lipase was fixed at 0.25 mg/mL, and the concentration of the substrate p‐NPB was changed (3, 4, 5, 6, 7 μmol/mL), and the enzyme reaction rates of zerofold, onefold, and twofold dilution were determined, respectively (using distilled water instead of zerofold dilution), and the experiments of each group were done three times in parallel. The Lineweaver‐Burk double inverse curve was plotted with the reciprocal of p‐NPB concentration as the horizontal coordinate and the reciprocal of the reaction rate as the vertical coordinate, and the type of lipase inhibition was judged according to the position of the intersection of the fitted straight line and the coordinate axis.

#### 2.2.3 Molecular Docking of Lipase With Proanthocyanidins

The protein file of pancreatic lipase (1LPA) was downloaded from Uniprot (https://www.uniprot.org/) and PDB (https://www.rcsb.org/) for molecular docking with proanthocyanidins.

### 2.3 Antioxidant Activity Studies

#### 2.3.1 ABTS Antioxidant Assay

Referring to the method in the literature with some modifications, 100 μL of each sample was taken and diluted 24, 25, 26, 27, 28, and 29 times. A total of 100 μL of ABTS (12 h reaction in the dark, diluted to near 0.7 absorbance when used) and ethanol were added in a 96‐well plate, and the absorbance was measured at 734 nm with VC (0.1 g/mL) as a positive control (Rašeta et al. 2024).

$$\text{ABTS free radical scavenging rate}\;\left(\%\right)=\left(1-\frac{A_{2}-A_{1}}{A_{0}}\right)\times 100$$

where *A*_0_: ABTS + ethanol (100 μL each) mixed well and absorbance at 734 nm; *A*_1_: Samples + ethanol (100 μL each) mixed well and absorbance at 734 nm; and *A*_2_: Samples + ABTS (100 μL each) mixed well and absorbance at 734 nm.

#### 2.3.2 DPPH Antioxidant Assay

Referring to the literature, the preparation of the DPPH reagent involved the following steps (Saadullah et al. 2024). First, 100 μL of each sample was taken, and the samples were then diluted 24, 25, 26, 27, 28, and 29 times. A total of 100 μL of DPPH (ethanol soluble) and 100 μL of ethanol were added to a 96‐well plate, and the absorbance was finally measured at 516 nm with VC (0.1 g/mL) as a positive control.

$$\text{DPPH free radical scavenging rate}\left(\%\right)=\left(1-\frac{A_{2}-A_{1}}{A_{0}}\right)\times 100$$

where *A*_0_: DPPH + ethanol (100 μL each) mixed well and absorbance at 516 nm; *A*_1_: Sample + ethanol (100 μL each) mixed well and absorbance at 516 nm; and *A*_2_: Samples + DPPH (100 μL each) mixed well and absorbance at 516 nm.

#### 2.3.3 Hydroxyl Radical Scavenging Antioxidant

Based on the literature, the sample solutions were first diluted 10, 30, and 50 times. Then, 1 mL of FeSO_4_ solution, 1 mL of salicylic acid‐ethanol solution, and 1 mL of H_2_O_2_ solution were added to the samples successively and mixed well (Haran et al. 2024). The reaction was conducted at 37°C for 30 min, and the absorbance was measured at 510 nm with VC (0.1 g/mL) as the positive control.

$$\text{Hydroxyl radical scavenging rate}\;\left(\%\right)=\frac{A_{0}-\left(A_{m}-A_{n}\right)}{A_{0}}\times 100$$

where *A*_*m*_: Absorbance of the sample at 510 nm; *A*_*n*_: Water was substituted for H_2_O_2_ with absorbance at 510 nm; and *A*_0_: Water instead of sample absorbance at 510 nm.

#### 2.3.4 Fe Ion Reduction Capacity Determination

The design of experimental protocols was improved in accordance with the literature. The sample solutions were diluted 5, 10, and 30 times. A total of 1 mL of phosphate buffer solution and 1 mL of 1% K_3_Fe (CN)_6_ solution were added to the sample successively, and the reaction was conducted under a water bath condition at 50°C for 20 min. After adding a 10% trichloroacetic acid solution, the sample was then centrifuged at 3000 r/min for 5 min (Wu et al. 2021). A total of 2 mL of supernatant was taken, 1 mL of water, and 1 mL of 0.1% FeCl_3_ solution were added, mixed, and left for 10 min, and the sample was analyzed. The absorbance was finally measured at 700 nm with VC (0.1 g/mL) as the positive control.

$$A\;\text{total antioxidant capacity}=A_{m}-A_{n}-A_{0}$$

where *A*_*m*_: Absorbance of the sample at 700 nm; *A*_*n*_: Absorbance at 700 nm for samples, buffers, and water; and *A*_0_: Absorbance at 700 nm for water substitution samples.

#### 2.3.5 Assay of Phosphomolybdic Acid Method

Referring to the literature method (Wang et al. 2022), the sample solutions were diluted 5, 10, and 30 times. A total of 2 mL of H_2_SO_4_ solution, 2 mL of phosphate solution, and 2 mL of ammonium molybdate solution were added to the samples successively and then mixed well. The solution was placed in a water bath at 95°C for 90 min and cooled to room temperature. Absorbance was measured at 760 nm with VC (0.1 g/mL) as a positive control.

$$A\;\text{total antioxidant capacity}=A_{m}-A_{n}-A_{0}$$

where *A*_*m*_: Absorbance of the sample at 760 nm; *A*_*n*_: Samples and absorbance at 760 nm for H_2_SO_4_ and phosphate and water; and *A*_0_: Absorbance at 760 nm for water substitution samples.

### 2.4 Total Ingredient Content

#### 2.4.1 Total Phenol Content

The total phenolic content (TPC) was determined by the Folin–Ciocalteu (FC) method (Vázquez et al. 2015). The standard curve of gallic acid (0.05 mg/mL) was first prepared in the concentration range of 1–6 mg/L. The FC reagent and 7.5% Na_2_CO_3_ solution were added after the samples were diluted well beforehand (1000‐fold dilution was optimal according to the pre‐experiment). The reaction was then conducted for 30 min under low light, and the absorbance at 760 nm was measured. The TPC of the wine samples was finally expressed as gallic acid equivalents.

#### 2.4.2 Total Flavonoid Content

Total flavonoid content (TFC) was determined by the NaNO_2_‐Al (NO_3_)_3_‐NaOH colorimetric method. The standard curve of rutin (0.5 mg/mL) was first prepared in the concentration range of 20–70 mg/L. After diluting the wine 50 times, 1 mL of 4% NaNO_2_ solution, 1 mL of 10% Al (NO_3_)_3_ solution, and 1 mL of 4% NaOH solution were added sequentially, with an interval of 6 min for each reagent. The reaction was finally realized after adding 60% vol. ethanol for 12 min. The supernatant was taken by centrifugation, and the absorbance at 510 nm was measured (Yao et al. 2016). The TFC in the wine samples was indicated as rutin equivalents.

#### 2.4.3 Total Proanthocyanin Content

The total anthocyanin content (TAC) was determined using the iron salt‐catalyzed colorimetric method. The standard curve of proanthocyanidins (0.5 mg/mL) was first prepared in the concentration range of 27–135 mg/L. The absorbance at 550 nm was then measured by adding ammonium iron sulfate and an n‐butanol hydrochloride solution to the wine samples diluted 75 times in advance, followed by heating at 95°C for 40 min (Pandeya et al. 2018). The total proanthocyanin content in the wine samples was finally expressed as proanthocyanidin equivalents.

### 2.5 UPLC‐Q‐Orbitrap‐MS Qualitative and Quantitative Chemical Composition

Q Exactive HF‐X Mass Spectrometer (Thermo, USA), Vanquish Ultra High‐Pressure Liquid Chromatograph (Thermo, USA), Chromatographic column: Waters HSS T3 (100 × 2.1 mm, 1.8 μm), Cryogenic high‐speed centrifuge (Eppendorf 5430R). Methanol/acetonitrile/formic acid/isopropanol (chromatography grade).

#### 2.5.1 Pretreatment

The wine samples were slowly thawed at 4°C, an appropriate volume of sample (0.5–1.0 mL, freeze‐drying and concentration can be used if the sample volume is too large) was taken into a centrifuge tube, two times the volume of extraction solution (methanol/acetonitrile, 1:1, v/v) was added, vortexed for 60 s, and ultrasonic extracted at low temperature for 30 min, and then the extract was centrifuged at 12000 rpm for 10 min at 4°C. The supernatant was then collected and placed at −20°C for 1 h to precipitate the protein, and then further centrifuged at 12,000 rpm for 10 min at 4°C; the supernatant was collected for vacuum drying, and 100 μL of 50% acetonitrile solution was added for re‐solubilization, and then centrifuged at 12000 rpm for 10 min at 4°C after vortexing, and then the supernatant was taken for on‐board assay.

#### 2.5.2 UPLC‐Q‐Orbitrap‐MS Parameters

Column: Waters HSS T3 (100 × 2.1 mm, 1.8 μm); mobile phase: ultra‐pure water (0.1% formic acid) in A, acetonitrile (0.1% formic acid) in B; flow rate: 0.3 mL/min; column temp: 40°C; injection volume: 2 μL; elution gradient: 0 min A‐phase/B‐phase (100:0, v/v), 12 min A‐phase/B‐phase 5% A‐phase, 95% A‐phase, 5% B‐phase, 13 min, 13.1% A‐phase, 86.9% B‐phase, 17 min, 100% A‐phase. The samples were kept at 4°C in the autosampler. To avoid fluctuations in the signal, a random sequence was used for the analysis of the samples. The QC method was used to monitor the system and data. A Q Exactive HFX from Thermo, USA, was used to obtain primary and secondary spectra. The instrument had an electrospray ionization (ESI) source with 40 arb of sheath gas and 10 arb of auxiliary gas, an ion spray voltage of +3000/−2800 V, a temperature of 350°C, and an ion transfer tube temperature of 320°C. The scanning mode was Full‐MS‐ddMS2, and the scanning mode was positive/negative ion. The mass spectrometry scanning range was 70–1050 Da, with a resolution of 70,000 and 17,500.

#### 2.5.3 Data Preprocessing

The data was processed using Progenesis QI software. This removed noise, identified peaks, matched peaks, corrected retention times, and aligned peaks. This created a data matrix with retention times, mass‐to‐charge ratios, and peak intensities. We used commercial databases and a database of secondary mass spectrometry of metabolites and corresponding cleavage patterns to identify the peaks containing secondary mass spectrometry data. The higher the secondary fragmentation score, the more reliable the identification result. A score of 0.7 or above is generally considered reliable. TICs for positive and negative ions were created with Xcalibur.

### 2.6 Network Pharmacological Study of Healthy Snow Lotus Wine

#### 2.6.1 Screening of Main Active Components and Target Prediction of Healthy Snow Lotus Wine

The compounds identified by UPLC‐Q‐Exactive Orbitrap‐MS were screened based on their relative content. The active ingredients were evaluated using Lipinski's rule. This means that the compounds had a molecular weight of less than 500, had no more than 5 hydrogen bond donors, and had no more than 10 acceptors. The number of hydrogen bond donors was not more than 10, and the log P was between −2 and 5 (Murugan et al. 2024). The screening process integrated data from the TCMSP database (Traditional Chinese Medicine Systems Pharmacology Database and Analysis Platform, TCMSP, https://tcmspw.com/index.php) and the SwissTargetPrediction database (http://www.swisstargetprediction.ch/). (Liu, Lu, et al. 2024; Liu, Ran, et al. 2024). Then, the active compounds' SMILES structures were determined using the PubChem database (https://pubchem.ncbi.nlm.nih.gov/). The SMILES structures of the active compounds were input into the SwissTargetPrediction database to predict their targets. The screening results were merged to eliminate duplicate entries. To identify disease targets associated with erectile dysfunction (ED), the OMIM database (Online Mendelian Inheritance in Man, OMIM, http://www.omim.org/), the GeneCards database (score ≥ 5) (https://www.genecards.org/), and the TTD database (Therapeutic Target Database, TTD, http://bidd.group/index.html/) were consulted. These targets were then combined and deduplicated to obtain disease targets.

#### 2.6.2 Network Diagram Construction

The Bioinformatics Platform (https://bioinformatics.com.cn/) was used to identify the intersections between disease targets and compound targets (Venn diagram). The resulting targets were imported into the STRING database (https://string‐db.org/) with a confidence score threshold set above 0.7. Unconnected targets were hidden, and interactions between targets were identified. The data were then imported into Cytoscape 3.10.0 software to construct a protein–protein interaction network diagram (Gong et al. 2023; Santos Sobrinho et al. 2022).

The chemical components of Healthy Snow Lotus Wine containing disease targets from the Venn diagram intersections were screened, and a drug‐component‐target network diagram was constructed. The more connections a component had with targets, the more critical it was.

#### 2.6.3 Enrichment Analysis

Kegg analyses were performed using the microbiology platform, and the analysis module integrated R packages such as clusterProfiler and pathview. Data were screened at *p*‐value ≤ 0.05, and GO analysis was applied to the first 10 entries, which were imported into the microbiology platform for visualization. KEGG was used for the first 10 pathways (Gajera et al. 2024).

#### 2.6.4 Docking of Molecules

Docking was done using LibDock in Discovery Studio 3.0. To find proteins, use the Uniprot database. Copy the PDB number and access the PDB database to download the 3D files of the desired proteins. Finally, use PubChem to find the 3D files of the core components. Next, open Discovery Studio (DS) software and remove water molecules, ligands, and complementary residues in preparation for docking. To process all compounds, select “Prepare Ligands” from the menu. Define each protein as a receptor molecule and use the machine learning module to predict binding regions. If the crystal structure does not include H atoms, hydrogenate the receptor before this step. Next, the processed compounds are docked with other compounds using the “Receptor‐Ligand Interactions” module. The results are then analyzed at the end of the program. The higher the docking score, the stronger the binding (Xiang et al. 2024).

## 3 Results

### 3.1 Results of Pancreatic Lipase Inhibition Assay

**FIGURE 1:**
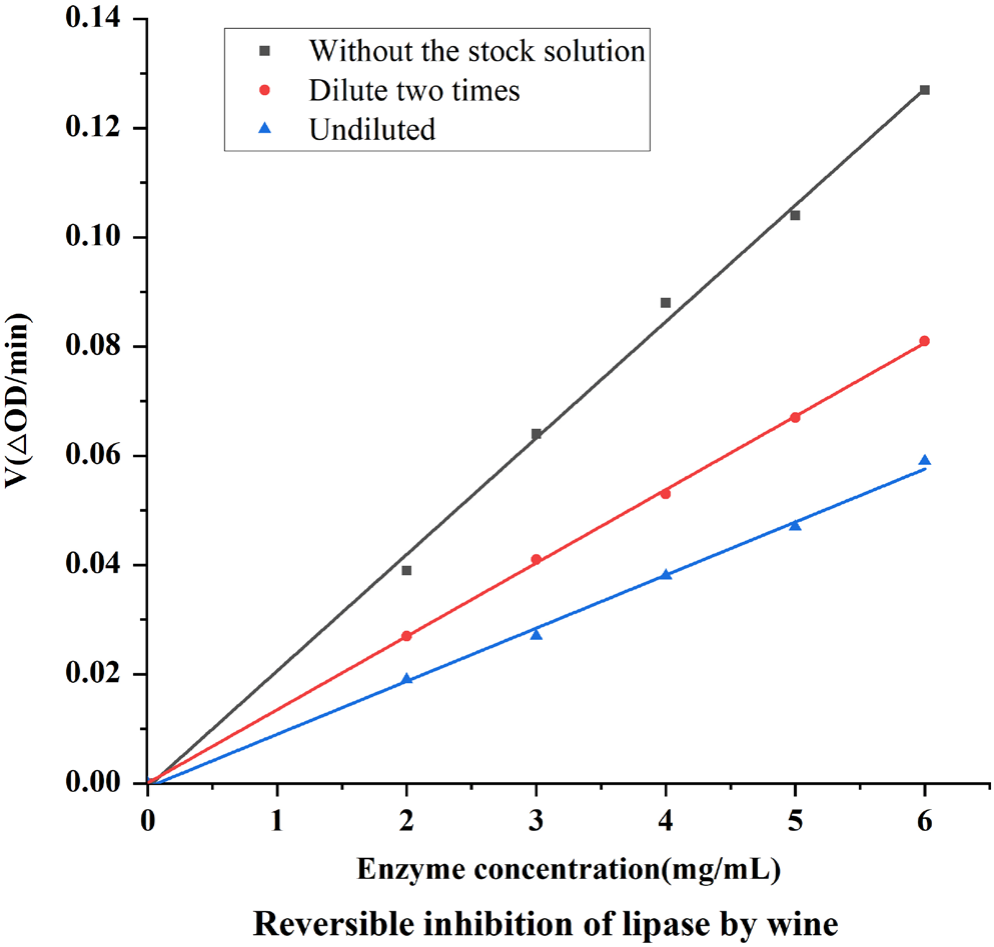
Reversible inhibition of lipase by red wine.

As can be seen from the Figure 1, the inhibitory effects of both gradually decreased with increasing dilution, but the inhibitory effect of wine decreased more rapidly, while orlistat inhibited lipase better than wine at all dilution levels. At dilutions ≥ 2, the inhibitory effect of wine decreased rapidly, whereas orlistat remained strong, suggesting that the lipase inhibitory effect of wine was limited at low concentrations, whereas orlistat remained effective at higher dilutions.

**FIGURE 2:**
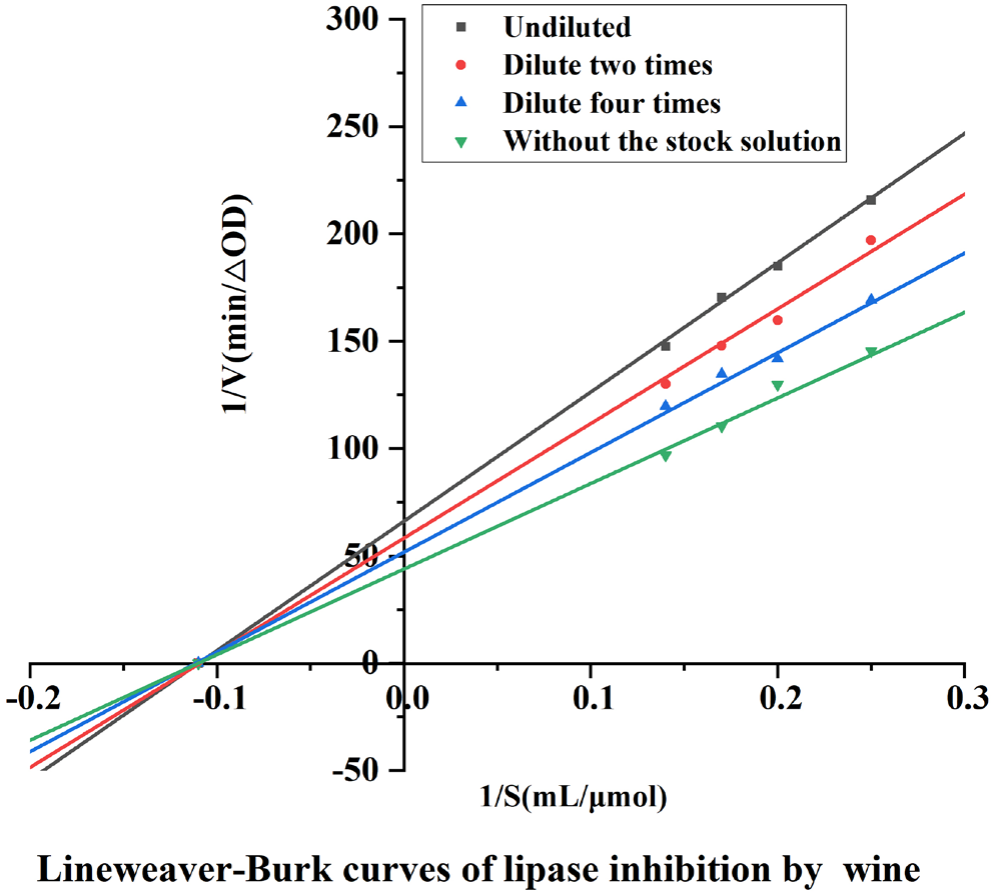
Lipase inhibition by red wine Lineweaver‐Burk curve.

The results of the kinetics of lipase inhibition by wine are shown in Figure 2, all the straight lines pass through the origin and the slopes increase with increasing wine dilution (i.e., decreasing wine concentration), which indicates that the inhibition of lipase by wine exhibits a concentration‐dependent effect, and it can be concluded that wine reversibly inhibits lipase activity.

Reversible inhibition is classified as non‐competitive, anti‐competitive, and competitive, and non‐competitive inhibition is typically characterized by unchanged Km and smaller Vmax. By plotting the Lineweaver‐Burk curves of the inhibitor group and the blank control group at different dilutions, it was found that the curves of the inhibitor group and the non‐inhibitor group intersected on the horizontal axis, and the intercept of the straight line on the horizontal axis remained unchanged, and the slopes became larger, i.e., the Km was unchanged and the Vmax became smaller, which showed that the type of inhibition of pancreatic lipase by wine was non‐competitive. lipase by wine is noncompetitive.

**FIGURE 3:**
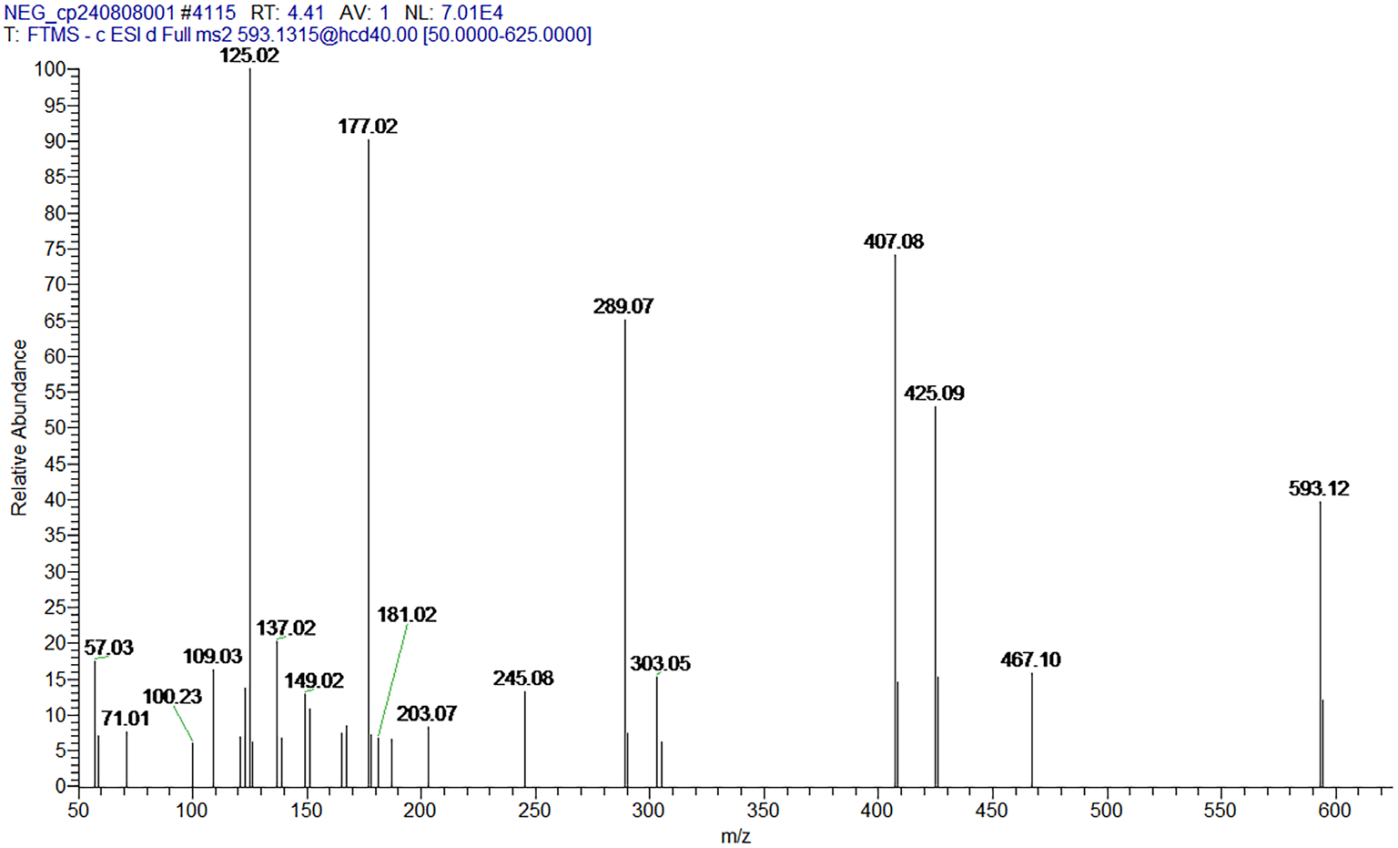
Secondary ion fragmentation identification of proanthocyanidins.

**FIGURE 4:**
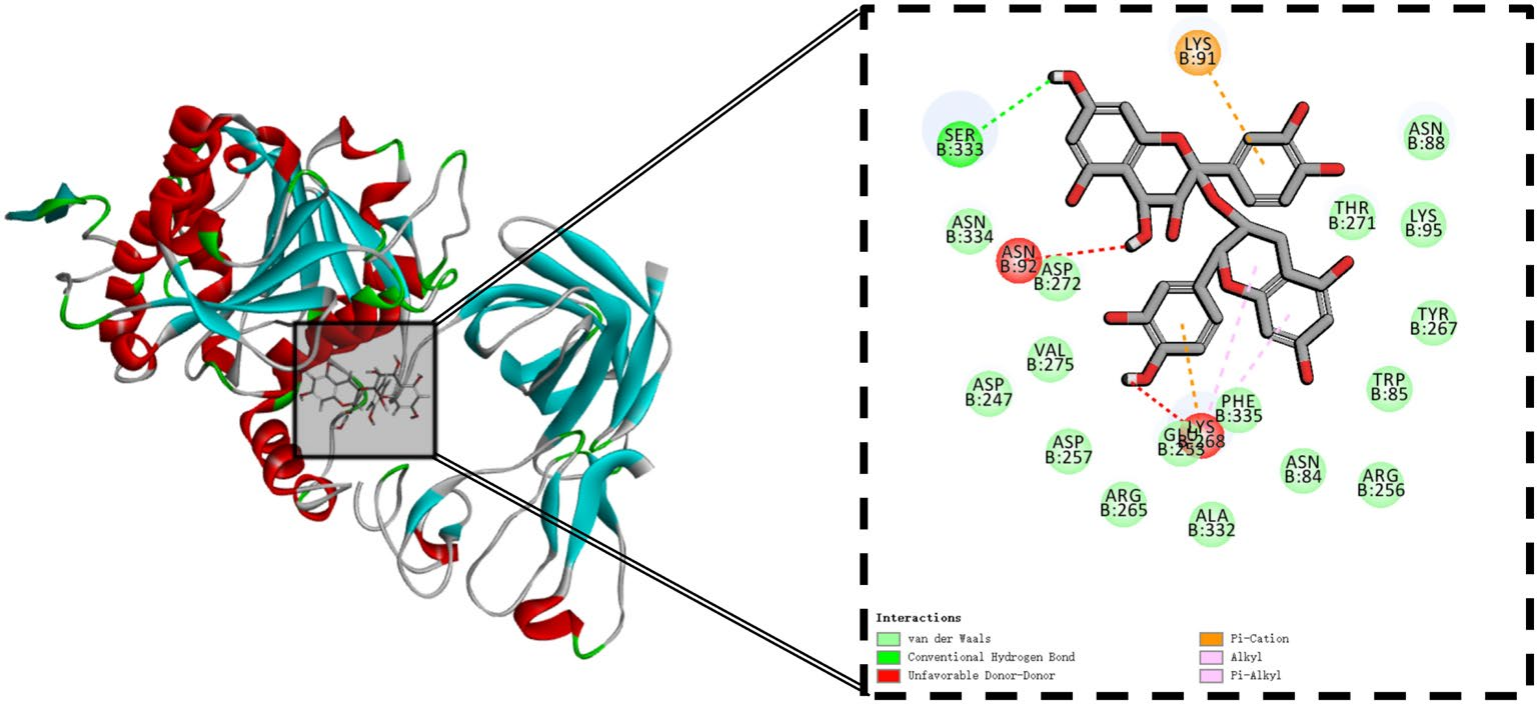
Molecular docking of proanthocyanidins with lipases.

The total ion flow map of procyanidins was obtained based on the mass spectrometry data, and the secondary fragmentation ions m/z 425, 407, 289, 245, 125 were identified (Xu et al. 2014). The results demonstrated concordance with the secondary mass spectra of procyanidins documented in the existing literature. Furthermore, molecular docking of procyanidins with pancreatic lipase was conducted, and the findings exhibited a notable multi‐targeting docking effect of procyanidin. As shown in Figures 3 and 4.

### 3.2 Antioxidant Capacity Test

TPC, an antioxidant, is also considered an important parameter for wine sensory characteristics. Therefore, several models of the most representative antioxidants were selected for the experiment: ABTS, DPPH, hydroxyl radical scavenging, ferric ion reducing capacity, and the phosphomolybdic acid method.

Fe^3+^ is formed into Fe^2+^ through antioxidants, and Fe^2+^ further reacts with FeCl_3_ and demonstrates maximum absorbance at 700 nm. Therefore, the absorbance at 700 nm can reflect the reduction capacity of antioxidants; that is, a large absorbance indicates a strong reduction capacity. The absorbance of W4 was significantly larger than the three remaining groups at three dilutions, second only to VC. This finding indicates that the effect of W4 on Fe^3+^ reduction was highly evident compared with the three remaining groups.

**FIGURE 5:**
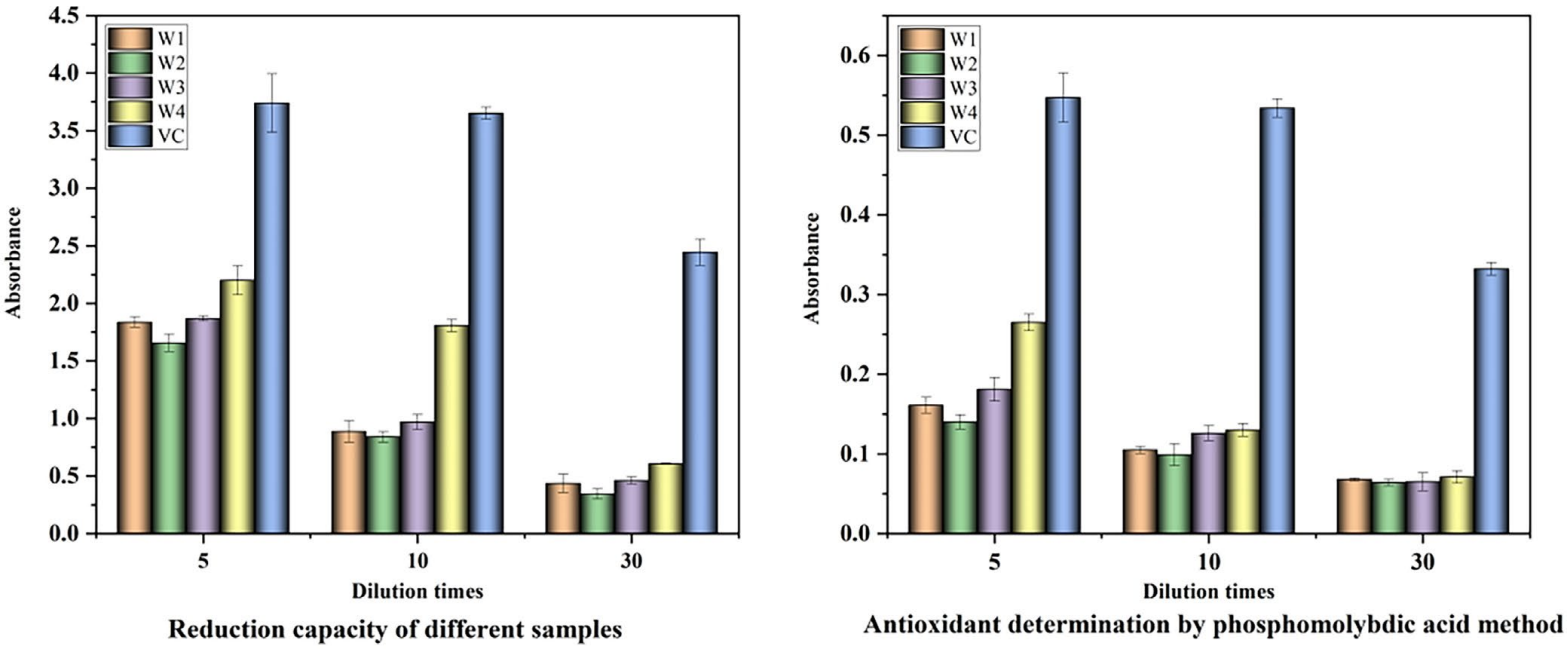
Results of reducing power of different samples and determination of reducing power of different samples of antioxidants by phosphomolybdic acid method.

Ammonium molybdate can produce blue compounds with antioxidants in the presence of strong acid and metaphosphate ions, and a high absorbance indicates strong antioxidant activity. The absorbance of W4 was significantly larger than the three other groups at all three dilutions, second only to VC, indicating that the total antioxidant capacity of W4 was stronger than the three other groups (Figure 5).

**FIGURE 6:**
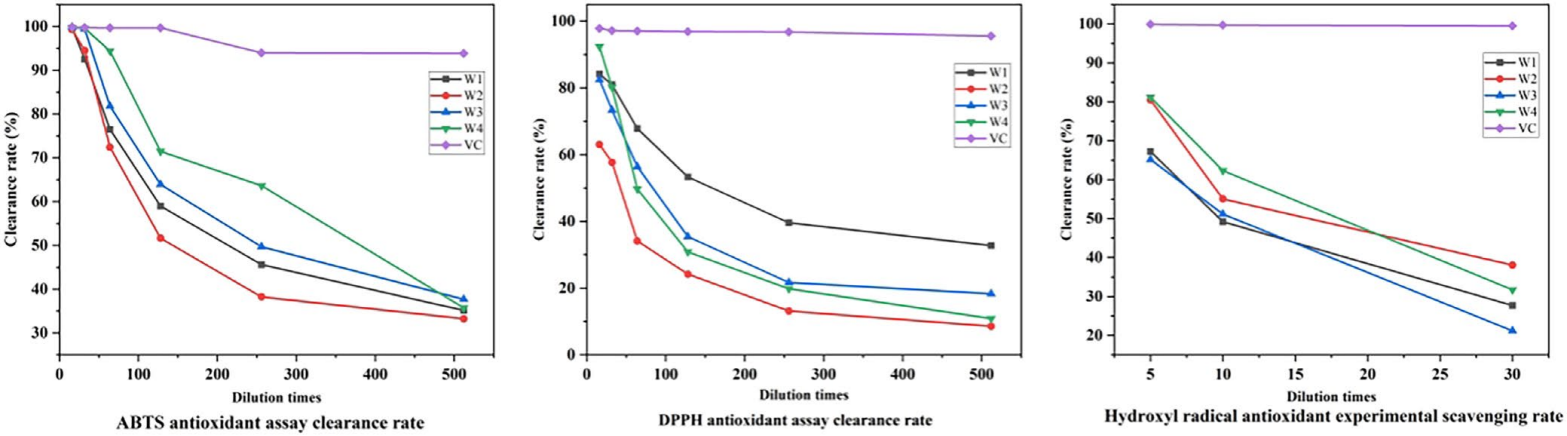
Detection of antioxidant capacity of ABTS, DPPH, and hydroxyl radicals.

ABTS was oxidized to green by the oxidant potassium persulfate, and the production of ABTS cationic radicals in the presence of the antioxidant was inhibited, resulting in a light solution color and a small absorbance value at 734 nm. The measured results are shown in Figure 6. W1, W2, W3, and W4 demonstrated strong scavenging activities against ABTS cationic radicals and a certain quantitative relationship with them. The scavenging rate was greater than 50% in the dilution range of 24–27, and only W4 had a scavenging rate greater than 50% at a dilution of 28, second only to VC.

In Figure 6, W1, W2, W3, and W4 showed strong scavenging activities against DPPH radicals and had a certain quantitative effect relationship with them. The scavenging rate was greater than 50% at the dilution range of 24–25, and the scavenging rates of W1 and W4 were close to and both higher than those of W2 and W3 at the dilution of 25. Meanwhile, the scavenging rate of W2 was always low.

The principle of hydroxyl radical scavenging lies in the generation of hydroxyl radicals by H_2_O_2_/Fe_2_^+^ through the Fenton reaction, which oxidizes Fe^2+^ to Fe^3+^ in the aqueous solution of o‐diazafil‐Fe^2+^, resulting in a decrease in absorbance at 510 nm. The capability of the sample to scavenge hydroxyl radicals is demonstrated by the decrease rate at 510 nm. The measured results are shown in Figure 6. W1, W2, W3, and W4 revealed strong scavenging activities for hydroxyl radicals and a certain quantitative effect relationship with them. Meanwhile, W4 was higher than the three remaining groups at dilutions of 5 and 10, second only to VC.

### 3.3 Determination of Total Content of Ingredients

The results in Table 1 were obtained from the experimental determination of total polyphenols in wines. The standard curves prepared before the determination of the total polyphenol and flavonoid contents were *y* = 123.31*x* + 0.0324, *R* ^2^ = 0.9991 and *y* = 12.76*x*−0.0172, *R* ^2^ = 0.9995, respectively. The TPC (365.7 ± 10.6 mg/100 mL) and TFC (176.3 ± 3.5 mg/100 mL) of Healthy Snow Lotus Wine were higher than those of the three other wines (*p* < 0.05). TPC was followed by W1 and W3 (both close in content) and then W2. The standard curve prior to the determination of proanthocyanidins was *y* = 5.1148*x* + 0.0627, *R* ^2^ = 0.9996. The proanthocyanidin content of the Healthy Snow Lotus Wine was significantly better than that of the other samples.

**TABLE 1:** Total phenolic content (TPC), total flavonoid content (TFC), and total proanthocyanidin content (TAC) of the wine samples.

Sample	Alcohol (%)	TPC (mg/100 mL)	TFC (mg/100 mL)	TAC (mg/100 mL)
W1	13.5	278.9 ± 2.6	113.7 ± 2.6	452.1 ± 10.2
W2	13.5	182.4 ± 3.3	105.2 ± 3.6	450.6 ± 9.0
W3	13.5	271.1 ± 1.3	175.8 ± 2.6	459.9 ± 4.5
W4	12.0	365.7 ± 10.6	176.3 ± 3.5	608.0 ± 2.2

*Note:* W4 is Healthy Snow Lotus Wine.

### 3.4 Results of UPLC‐Q‐Orbitrap‐MS

**FIGURE 7:**
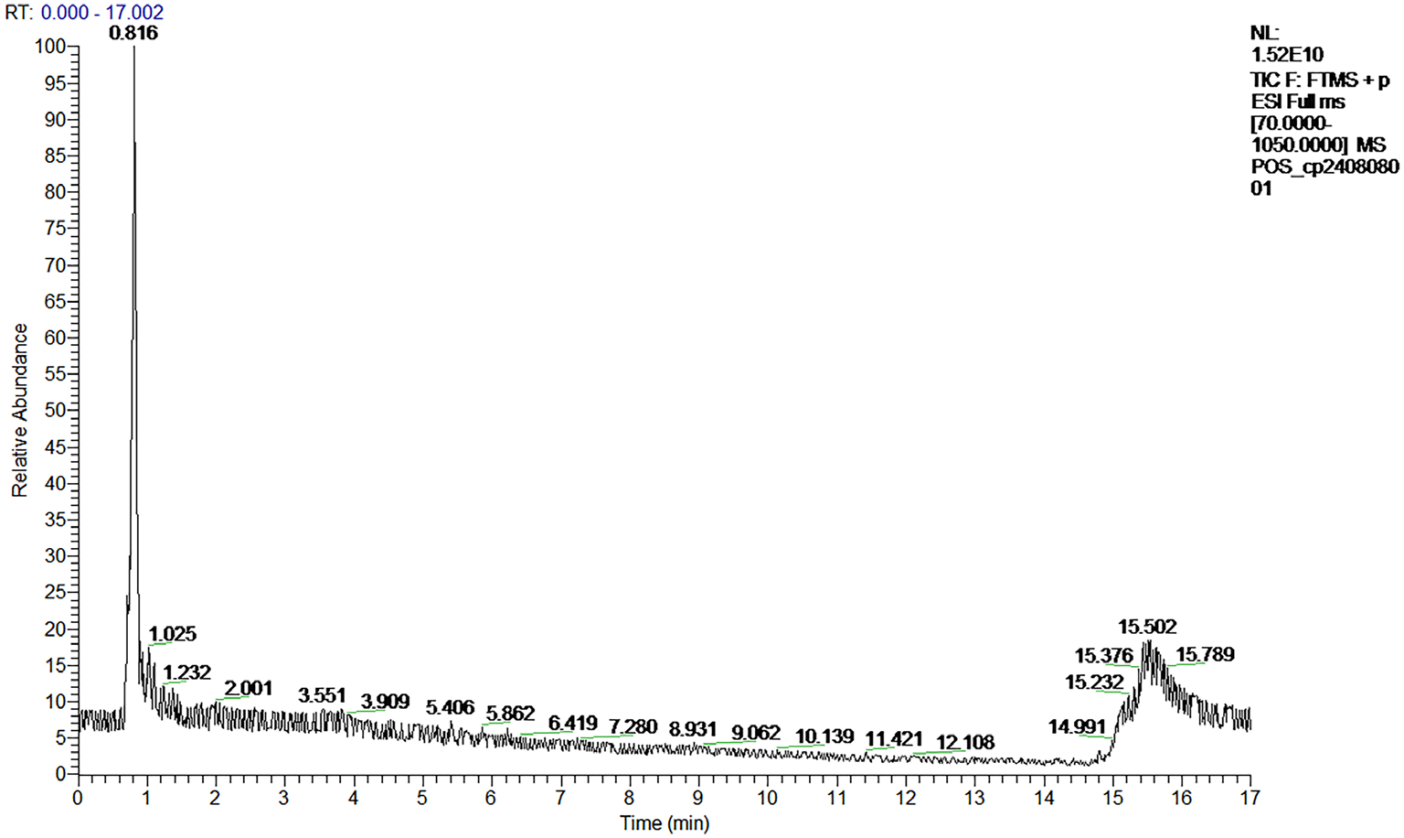
Total ion current diagram in positive ion mode.

**FIGURE 8:**
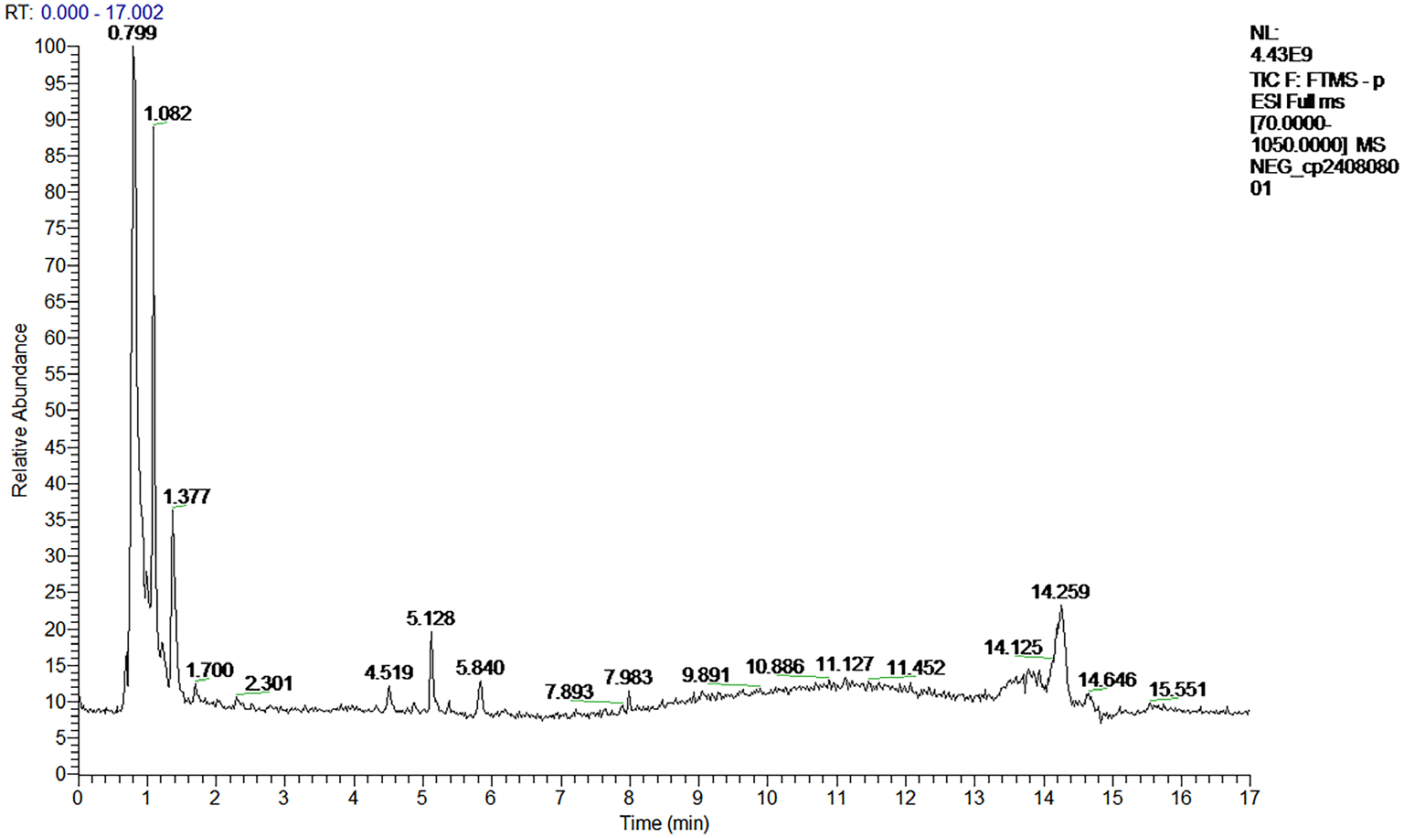
Total ion current diagram in negative ion mode.

The identification results were collated and screened for substances with a relative content > 0.2% and conforming to Lipinski's five‐fold rule, and the total ionic current diagrams in positive and negative modes are shown in Figures 7 and 8. Relevant data are shown in Table 2.

**TABLE 2:** UPLC‐Q‐Orbitrap‐MS data information.

No	Metabolite	Mode	Adducts	Formula	m/z	Fragment ions
1	D‐proline	pos	M + H, 2 M + H	C_5_H_9_NO_2_	116.070555	58.0658;59.0735;68.0499;69.7626;70.0656;70.3695;74.9377;98.0607;98.97;116.0705
2	Succinic acid	neg	M‐H_2_O‐H, M‐H	C_4_H_6_O_4_	117.019247	52.9735;54.3025;55.0187;73.0294;85.8585;94.3392;94.3506;99.0085;116.93;117.0192
3	L‐asparagine	pos	M + H	C_4_H_8_N_2_O_3_	133.060595	68.0498;70.029;74.0241;86.0604;87.0555;88.0399;99.0078;116.0337;132.0648;133.0583
4	1‐Heptanol	pos	2 M + ACN + H	C_7_H_16_O	274.273473	57.0707;62.0603;70.0657;71.0861;85.1017;88.076;102.0913;106.0867;256.262;274.2736
5	D‐leucine	pos	M + H	C_6_H_13_NO_2_	132.101764	69.0343;69.0704;72.0448;79.4133;86.0968;87.0442;91.0538;97.029;105.0698;132.1019
6	3‐O‐Debenzoylzeylenone	pos	2 M + Na	C_14_H_14_O_6_	579.148646	123.044;127.0388;135.0439;139.0384;163.0387;271.0601;275.0551;287.0546;289.0701;409.0901
7	5‐deoxy Thymidine	pos	M + CH_3_OH + H	C_10_H_14_N_2_O_4_	259.128355	56.05;84.0811;114.0545;122.0964;142.049;168.1016;170.0807;195.1122;213.1228;259.1284
8	Glycolaldehyde dimer	neg	M + FA‐H	C_4_H_8_O_4_	165.040644	57.0347;59.0138;72.993;75.0087;87.0087;99.009;105.0193;129.0192;147.0297;165.0406
9	Butyric acid	pos	M + ACN + H, 2 M + ACN + H	C_4_H_8_O_2_	130.086097	55.5351;56.0501;61.1989;70.0658;72.6917;75.4781;81.2637;84.0451;84.0811;130.0861
10	2‐Ketobutyric acid	neg	M + FA‐H	C_4_H_6_O_3_	147.030046	57.0346;59.0139;85.0293;87.0088;101.0242;103.0405;129.0204;129.0409;146.0253;147.0294
11	Ethyl gallate	neg	M‐H	C_9_H_10_O_5_	197.045798	111.0086;124.0167;125.0244;127.0036;136.9877;139.0036;140.0112;168.0067;169.0145;197.0458
12	Adenine	pos	M + H	C_5_H_5_N_5_	136.061577	91.0545;93.0696;94.0401;107.0615;107.0846;109.0758;119.0343;135.0916;136.0246;136.0608
13	(+)‐Catechin	neg	M‐H, M + Cl	C_15_H_14_O_6_	289.071736	59.0138;109.0294;123.0451;125.0244;137.0247;151.04;203.0717;205.0507;245.0821;289.0718
14	(S)‐b‐aminoisobutyric acid	pos	M + H‐H_2_O, M + H	C_4_H_9_NO_2_	104.070929	58.0658;59.0736;60.0813;67.2093;69.034;77.0948;86.0606;87.0445
15	4‐Hydroxyproline	pos	M + H	C_5_H_9_NO_3_	132.065392	55.0184;68.0499;69.0341;70.0657;74.0241;86.0604;86.0969;87.0544;97.0286;114.055
16	Cyclic N‐Acetyl‐D‐mannosamine	neg	M + FA‐H	C_8_H_15_NO_6_	266.087992	59.0138;72.0091;74.0248;84.0451;101.0245;104.0354;146.0459;176.0568;218.0667;266.0891
17	Ureidopropionic acid	neg	M‐H_2_O‐H, M‐H	C_4_H_8_N_2_O_3_	131.046232	58.0298;70.0299;71.0137;72.0092;88.0404;95.025;113.0355;114.0196;115.0029;131.0468
18	Trilepisflavan	neg	M + Cl	C_34_H_34_O_8_	605.194083	59.0137;71.0137;85.0294;87.0084;89.0242;101.0241;113.0242;151.0614;173.0455;605.1937
19	D‐serine	pos	M + H	C_3_H_7_NO_3_	106.050062	58.0658;60.0449;60.0759;60.0813;61.0484;61.0846;70.0294;88.0393;105.1107;106.05
20	L‐glutamic acid	neg	M‐H_2_O‐H, M‐H	C_5_H_9_NO_4_	146.045967	102.0559;128.0353;130.5652;146.046
21	(−)‐Epicatechin	neg	M‐H, M + Cl	C_15_H_14_O_6_	289.071738	109.0293;123.045;125.0243;137.0243;151.0401;179.035;203.0715;205.0506;245.0818;289.0718
22	quercetin	pos	M + H	C_15_H_10_O_7_	303.049302	121.0284;137.0229;153.0179;165.0181;201.0545;229.0489;257.0442;274.0457;285.0395;303.0491
23	Clostebol acetate	neg	M + K‐2H	C_21_H_29_ClO_3_	401.130586	59.0137;71.0137;89.0243;101.0243;113.0241;119.0346;161.045;179.0565;221.0681;341.1106
24	De‐O‐methyllasiodiplodin	neg	M‐H	C_16_H_22_O_4_	277.144601	107.0503;121.0294;127.1127;129.1286;134.0373;134.8947;147.0084;205.1599;233.1543;277.1445
25	Sucrose	neg	M + FA‐H	C_12_H_22_O_11_	387.114691	59.0138;71.0137;72.9934;89.0243;101.0244;113.0243;119.0343;179.0566;193.0354;341.1096
26	L‐aspartic acid	pos	M + H	C_4_H_7_NO_4_	134.044600	70.029;70.0659;71.0495;74.024;74.0335;87.055;88.0395;88.076;116.0335;134.0431
27	Apigenin	pos	M + H	C_15_H_10_O_5_	271.059414	67.0183;68.9974;91.0547;119.0491;121.0282;145.0282;153.0179;163.0397;270.7722;271.0592
28	Cyclo (Ile‐Leu)	neg	2 M + Hac‐H	C_12_H_22_N_2_O_2_	511.350013	59.0138;112.0773;129.1043;130.0868;207.1495;224.1743;225.1597;337.2609;433.3187;451.3295

### 3.5 Study on the Mechanism of Intervention in Erectile Dysfunction by Healthy Snow Lotus Wine

#### 3.5.1 Target Prediction and Network Diagram Construction

**FIGURE 9:**
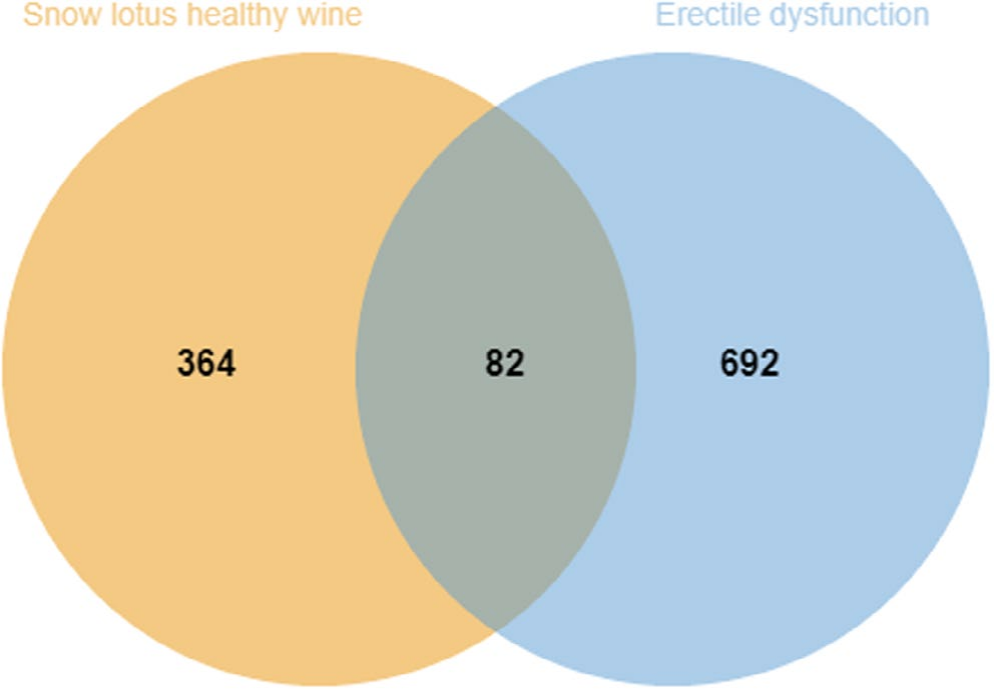
Erectile dysfunction and Healthy Snow Lotus Wine target Venn diagram.

**FIGURE 10:**
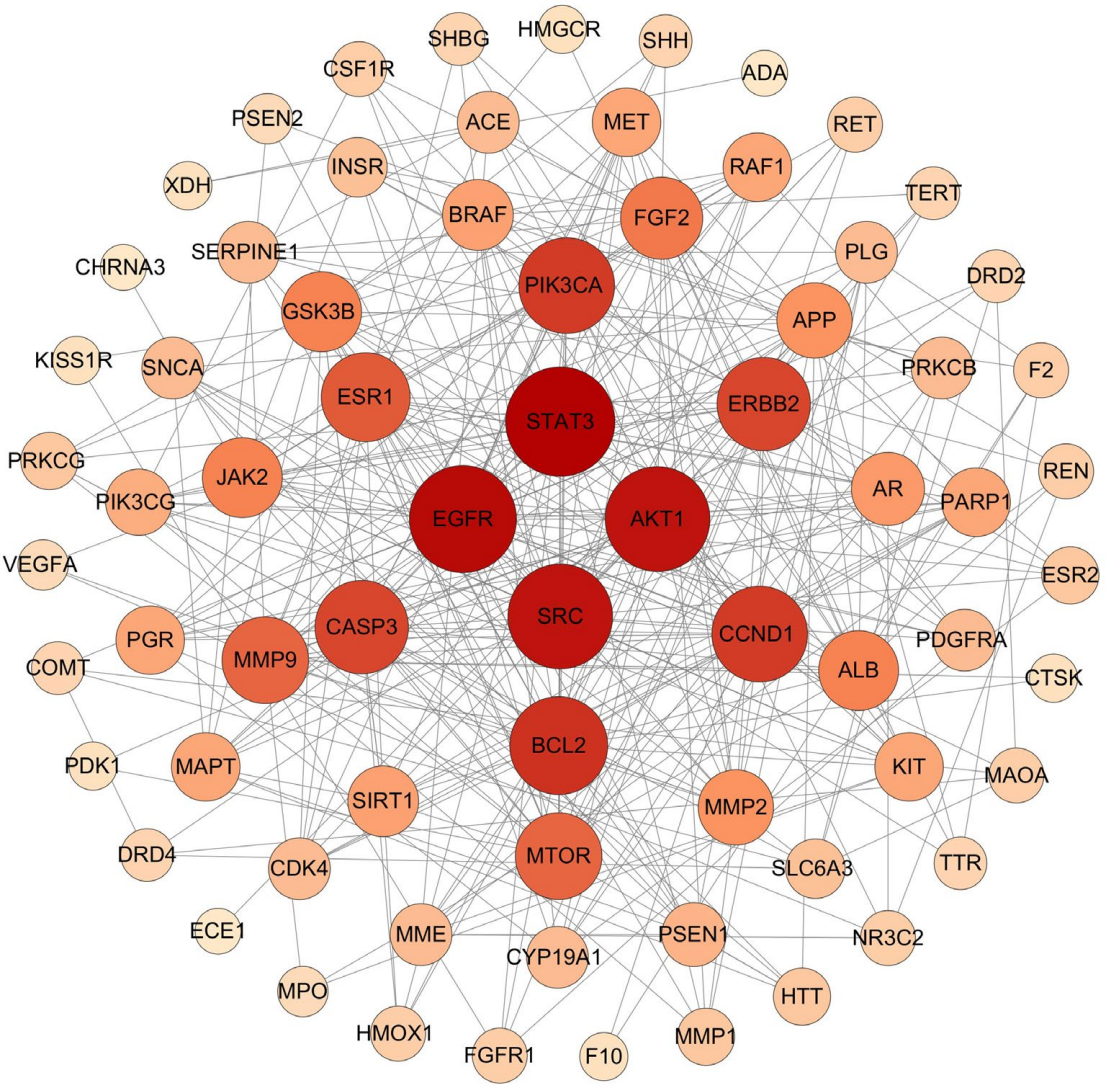
Protein–protein interaction networks.

**FIGURE 11:**
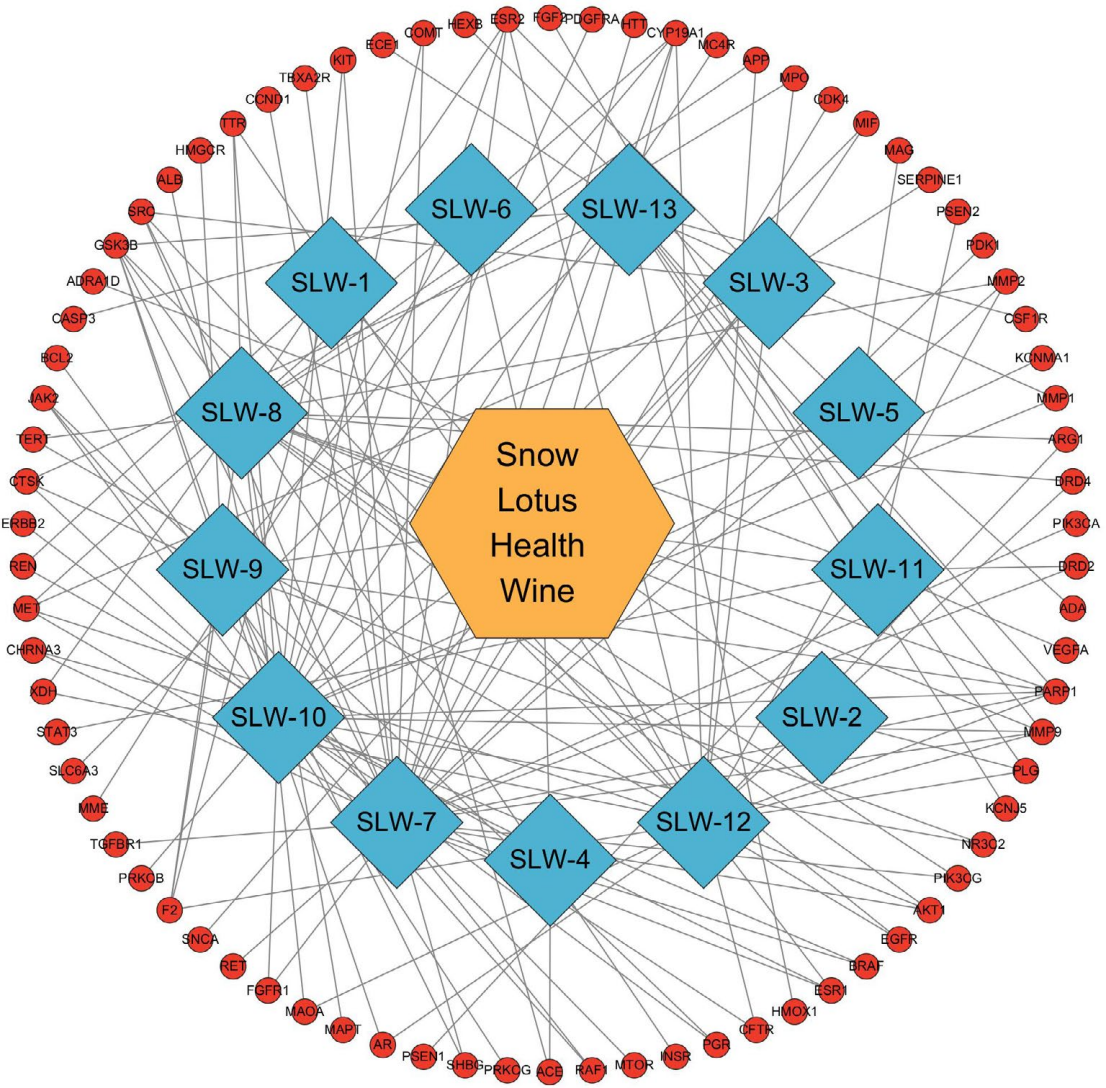
Component target interaction network.

Based on the screening criteria described in Section 2.3.1 (including Lipinski's rule), 28 compounds met the criteria, with 774 intersections between disease targets and 82 intersections between targets. Figure 9 shows the Wayne diagram. Figure 10 shows the protein–protein interaction network. It was made using the criteria in the previous section. Figure 11 shows the components that match Lipinski's law of five‐fold multiplicity. Table 3 lists the active ingredients.

**TABLE 3:** Key active ingredients of *Anchusa italica* Retz. flowers.

ID	Metabolite	Pubchem_ID
SLW‐1	D‐proline	8988
SLW‐2	D‐leucine	439524
SLW‐3	Ethyl gallate	13250
SLW‐4	4‐Hydroxyproline	825
SLW‐5	Cyclic N‐Acetyl‐D‐mannosamine	11096158
SLW‐6	Ureidopropionic acid	111
SLW‐7	Trilepisflavan	131848132
SLW‐8	Quercetin	5280343
SLW‐9	Clostebol acetate	13327
SLW‐10	De‐O‐methyllasiodiplodin	14562695
SLW‐11	Sucrose	5988
SLW‐12	Apigenin	5280443
SLW‐13	Cyclo (Ile‐Leu)	63103176

The four substances most closely linked to each target were Trilepisflavan, De‐O‐methyllasiodiplodin, apigenin, and quercetin. The degree values are 32, 27, 23, 13.

#### 3.5.2 Results of GO and Kegg Enrichment Analysis

**FIGURE 12:**
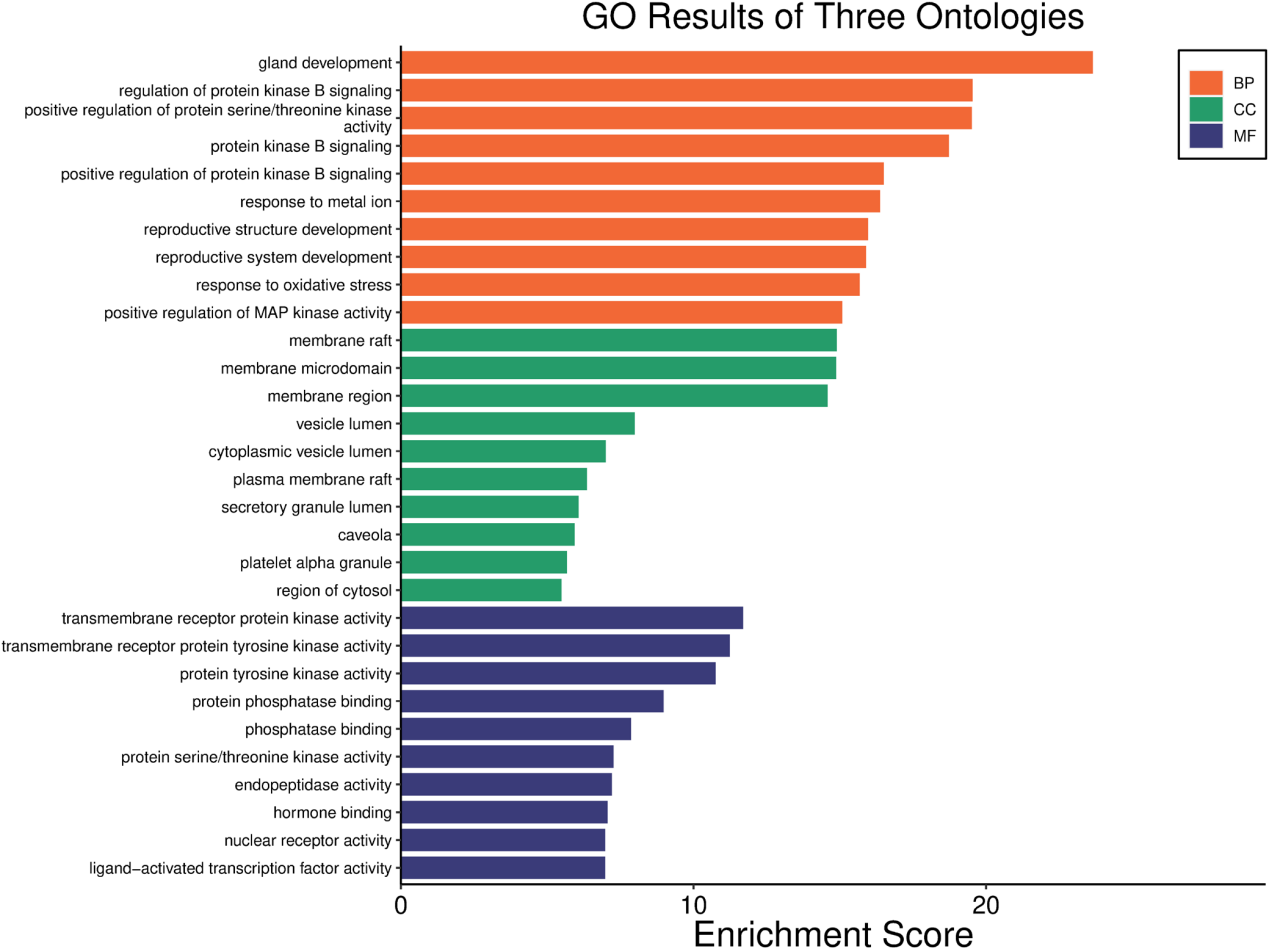
BP, CC, MF, 3‐in‐1 enrichment results.

**FIGURE 13:**
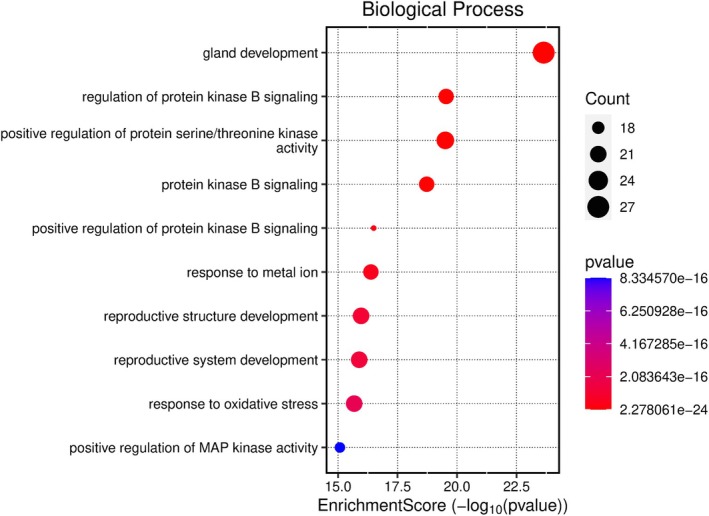
BP‐Enrichment Score.

**FIGURE 14:**
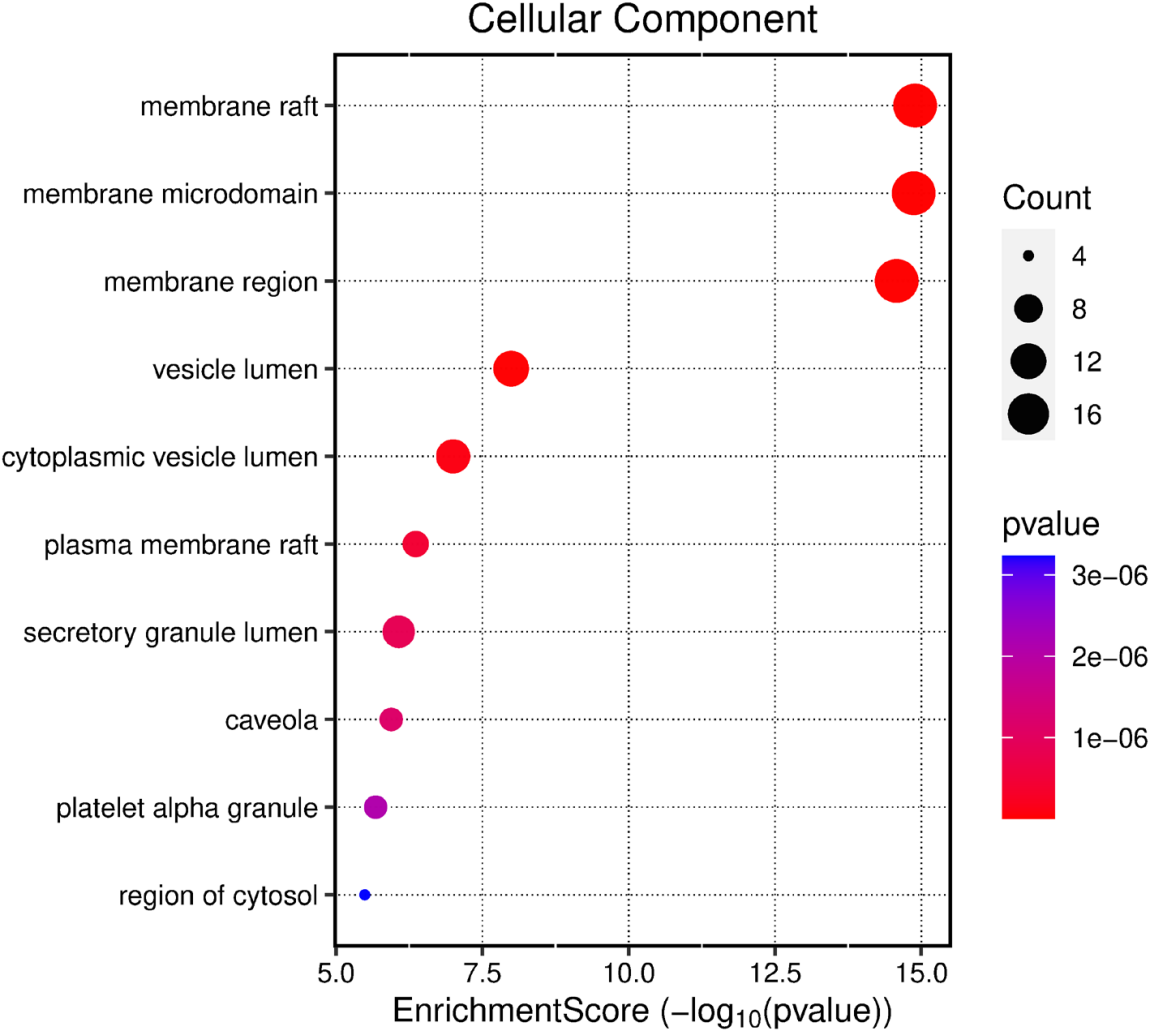
CC‐Enrichment Score.

**FIGURE 15:**
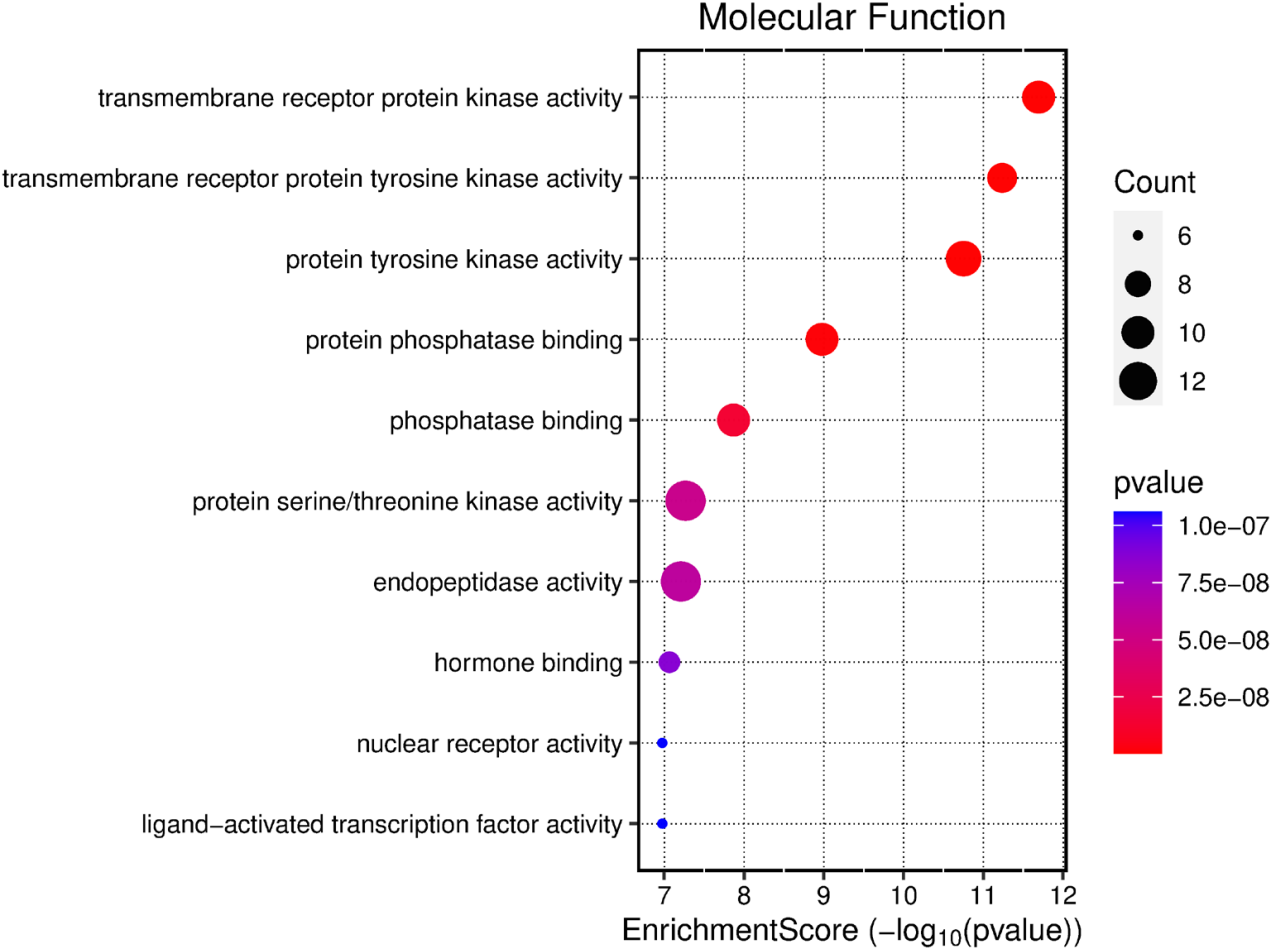
MF‐Enrichment Score.

**FIGURE 16:**
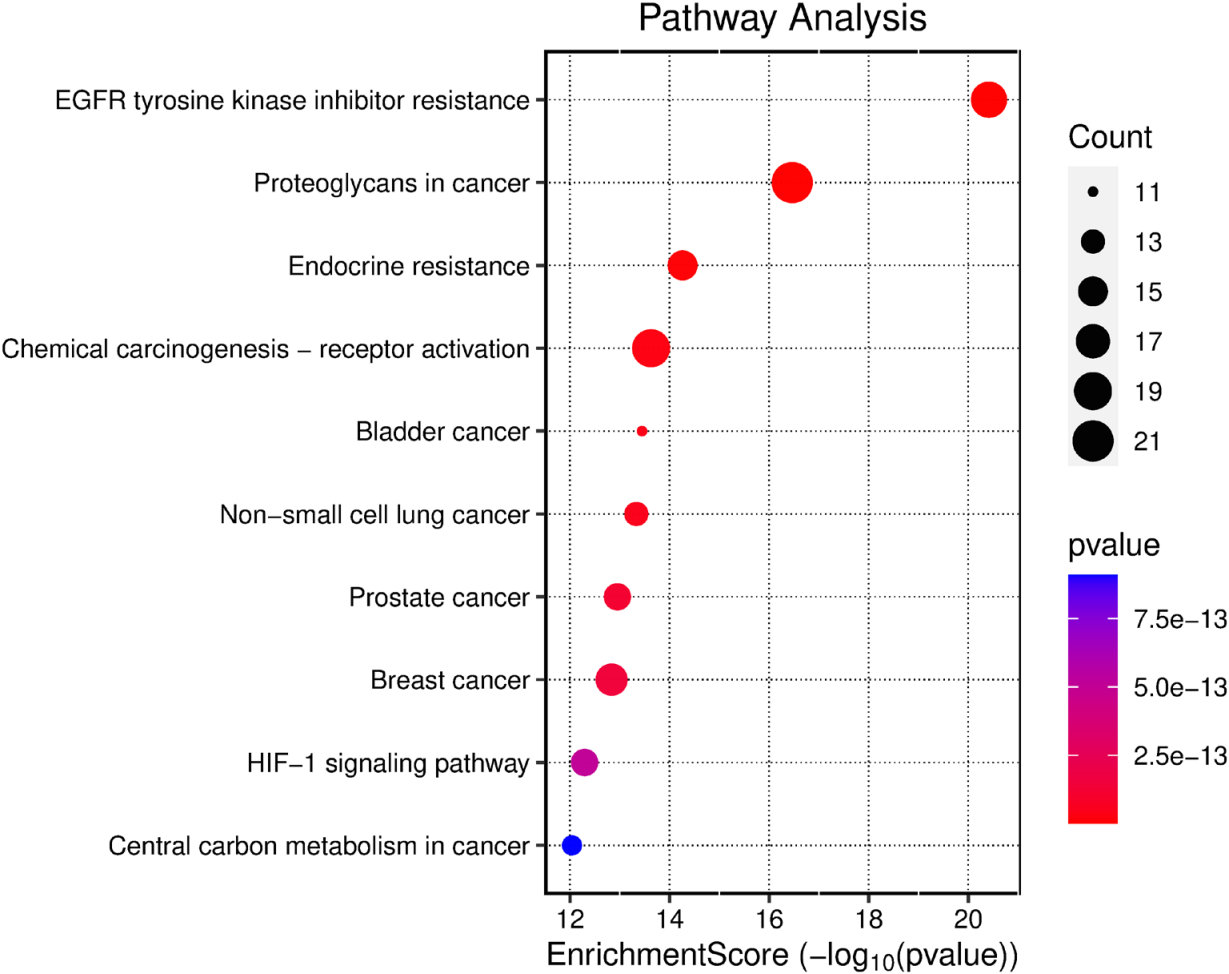
KEGG enrichment analysis results.

Kegg analyses were performed using the microbiology platform and the analysis module integrated R packages such as clusterProfiler and pathview. The data were screened at a *p*‐value of ≤ 0.05, and 2179 BP entries were obtained, mainly related to chemical stress, signaling, and stress response. A total of 107 CC entries were identified, mainly related to the cell basement membrane and phosphatidylinositol 3‐kinase complex. A total of 131 MF entries were identified, mainly involving phosphatase binding, phosphatidylinositol kinase activity, and protein phosphatase binding. The top 10 entries of each item were imported into the microbial platform for visualization. The GO enrichment analysis results are shown in Figures 12, 13, 14, 15. One hundred and fifty pathways were identified by KEGG screening, and the top 10 pathways were analyzed. The bubble diagram is shown in Figure 16.

#### 3.5.3 Docking of Molecules

The top three‐degree components were docked with the top five‐degree proteins. Core proteins are shown in Table 4.

**TABLE 4:** Core protein‐related information.

ID	Uniprot ID	PDB ID	Degree
STAT3	P40763	6NJS	29
EGFR	P00533	8a27	28
SRC	P12931	1FMK	27
AKT1	Q38998	5AAR	27
BCL2	Q07820	8VJP	24

**FIGURE 17:**
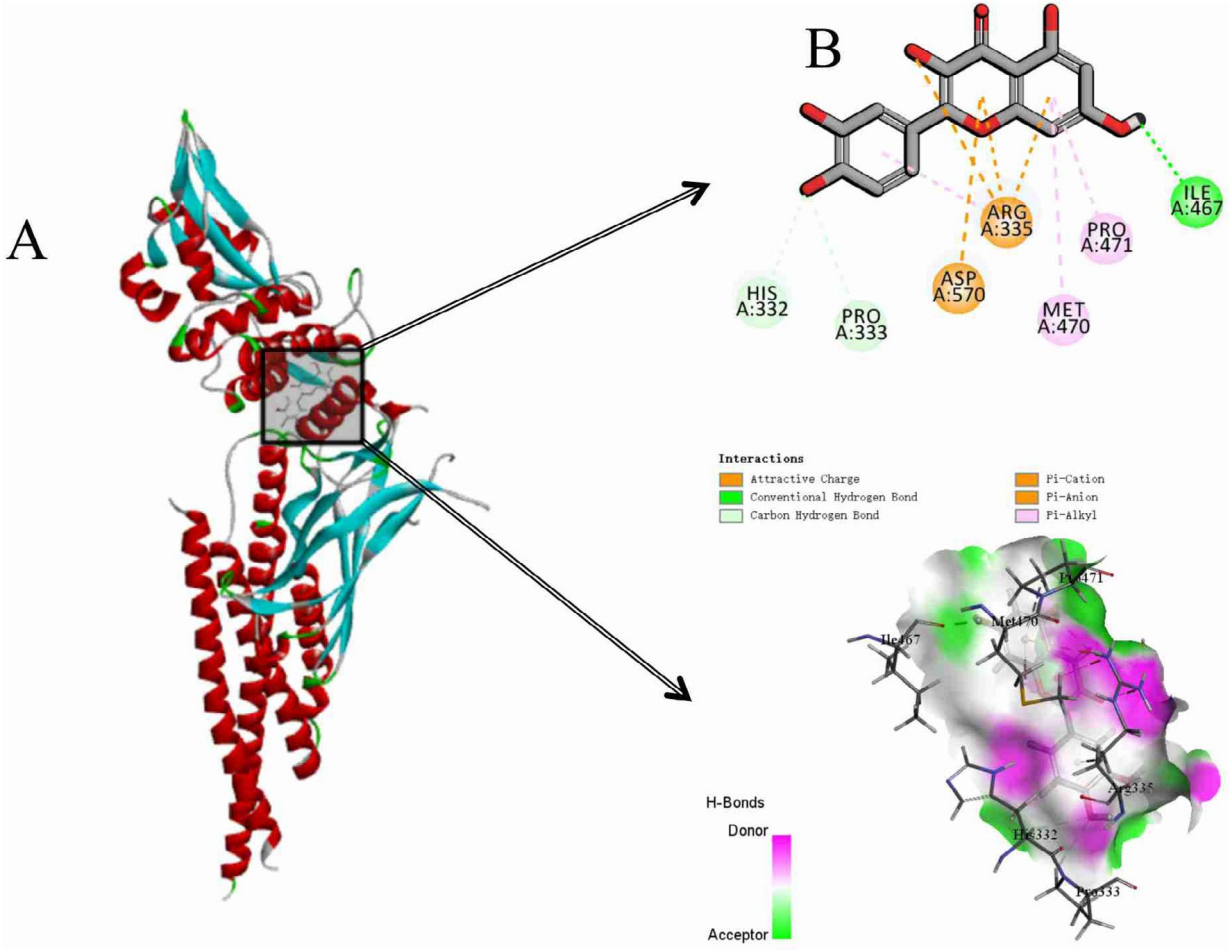
Molecular docking results of quercetin with STAT3 (A: overall docking results, B: 2D and 3D display of molecule‐specific docking points).

**FIGURE 18:**
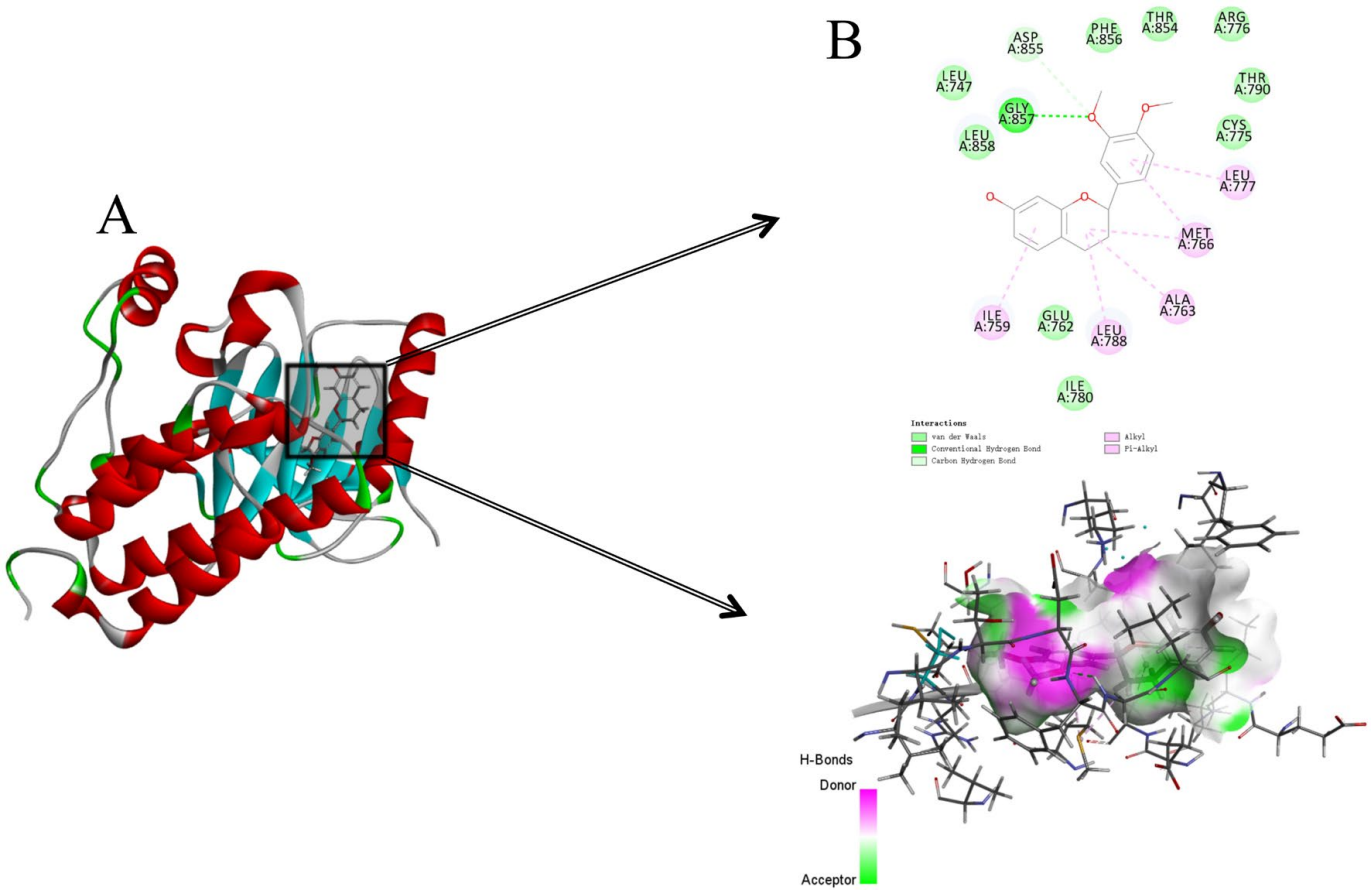
Molecular docking results of Trilepisflavan and EGFR (A: overall docking results, B: 2D and 3D display of molecule specific docking sites).

STAT3 is a key signal transducer and transcriptional activator involved in multiple cytokine signaling (Zhao et al. 2021). In the context of erectile dysfunction (ED), STAT3 may act by affecting vascular endothelial function and smooth muscle cell proliferation and apoptosis. EGFR is a transmembrane tyrosine kinase involved in the regulation of cell proliferation, differentiation, and survival. In the context of ED, EGFR may affect erectile function by regulating angiogenesis and tissue repair processes in the penile corpus cavernosum (Masuda et al. 2023). SRC is a non‐receptor tyrosine kinase involved in multiple signaling pathways (Hussain et al. 2023). SRC may play a role in regulating vascular tone and influencing the contraction of penile corpus cavernosum smooth muscle, which in turn affects erectile function. AKT1 is a key molecule in the PI3K‐AKT signaling pathway and is involved in processes such as cell survival, proliferation, and neovascularization (Fang et al. 2019). In ED, AKT1 may play a role by affecting vascular endothelial function and smooth muscle cell apoptosis, which in turn affects erectile function. BCL2 is an anti‐apoptotic protein that regulates cell survival by inhibiting apoptosis. In the context of ED, BCL2 may act by affecting the apoptotic process in penile cavernous tissue, especially in diabetes‐related ED, and BCL2 may protect cavernous tissue through its anti‐apoptotic effects (Shit et al. 2024). Molecular docking of quercetin with STAT3 as well as rilepisflavan and EGFR showed the best results, as shown in Figures 17 and 18.

## 4 Discussion and Conclusion

By employing the UPLC‐Q‐Orbitrap‐MS technique, we were able to identify a variety of metabolites in Healthy Snow Lotus Wine that complied with Lipinski's five rules (Karami et al. 2022). These metabolites may possess biological activity. The constructed protein interaction network and enrichment analysis demonstrated that Healthy Snow Lotus Wine was significantly associated with chemical stress, signal transduction pathways, and stress response pathways, particularly at the cellular basement membrane and phosphatidylinositol 3‐kinase complex (Jędrzejewski et al. 2025). This finding offers insight into the potential therapeutic intervention effect of Healthy Snow Lotus Wine on erectile dysfunction. With regard to the testing of bioactivity, Healthy Snow Lotus Wine demonstrated reversible noncompetitive lipase inhibition (Wang et al. 2024). Antioxidant experiments revealed that Healthy Snow Lotus Wine exhibited a more robust antioxidant capacity than the other samples, accompanied by a more pronounced total phenolic content (TPC), total flavonoid content (TFC), and total proanthocyanidin content (TAC). The present study reveals the potential mechanism by which Healthy Snow Lotus Wine intervenes in erectile dysfunction (ED) by acting on multiple biological targets. This is consistent with the use of Snow Lotus as a pleiotropic therapeutic agent in traditional medicine. The active ingredients in Healthy Snow Lotus Wine may exert a beneficial effect on ED by protecting vascular endothelial function and reducing oxidative stress, due to their potent antioxidant and anti‐inflammatory effects. The Healthy Snow Lotus Wine has been shown to assist in the regulation of fat metabolism by reversibly and noncompetitively inhibiting lipases, thereby reducing the risk of obesity and metabolic syndrome, which in turn has a positive effect on the prevention of erectile dysfunction (ED). In addition, the multicomponent properties of Healthy Snow Lotus Wine suggest potential benefits in the prevention of cardiovascular disease and anti‐tumor effects. However, further research is required to comprehensively assess the pharmacological effects, pharmacokinetic properties, bioavailability, and safety of its active constituents. Future studies should focus on the specific mechanism of action of these active ingredients in the clinical treatment of ED as well as their metabolic process and safety evaluation in vivo. Concurrently, further clinical trials will facilitate the validation of existing studies and furnish a scientific basis for the utilization of Healthy Snow Lotus Wine as an adjuvant treatment for ED.

## Author Contributions

**Bohan Yang:** conceptualization (lead), data curation (equal), formal analysis (equal), investigation (lead), methodology (equal), writing – original draft (equal), writing – review and editing (equal). **Siyu Tao:** methodology (equal), project administration (equal), supervision (lead), writing – original draft (equal). **Linyang Wang:** data curation (supporting), validation (equal). **Yanna Yao:** data curation (equal), validation (equal). **Xinyu Wang:** visualization (equal), writing – original draft (supporting), writing – review and editing (supporting). **Shuge Tian:** visualization (equal), writing – original draft (supporting), writing – review and editing (supporting).

## Consent

The authors have nothing to report.

## Conflicts of Interest

The authors declare no conflicts of interest.

## Data Availability

The original contributions presented in the study are included in the article; further inquiries can be directed to the corresponding author.
